# Nucleus Accumbens Volume Is Associated with Frequency of Alcohol Use among Juvenile Justice-Involved Adolescents

**DOI:** 10.3390/brainsci2040605

**Published:** 2012-11-12

**Authors:** Rachel E. Thayer, Shirley M. Crotwell, Tiffany J. Callahan, Kent E. Hutchison, Angela D. Bryan

**Affiliations:** 1 Department of Psychology and Neuroscience, University of Colorado Boulder, Muenzinger D244, UCB 345, Boulder, CO 80309-0345, USA; Email: tiffany.callahan@colorado.edu (T.J.C.); kent.hutchison@colorado.edu (K.E.H.); angela.bryan@colorado.edu (A.D.B.); 2 Department of Psychology, University of New Mexico, MSC03 2220, Albuquerque, NM 87131-0001, USA; Email: ssmith3@unm.edu

**Keywords:** adolescence, alcohol use, nucleus accumbens, orbitofrontal cortex, juvenile justice

## Abstract

Differential neural development of structures associated with reward and control systems may underlie risky behavior in adolescence. The nucleus accumbens and orbitofrontal cortex (OFC) have been implicated in substance use behavior, although structural studies have yet to explore specific relationships between nucleus accumbens and OFC volumes and alcohol use in adolescence. High resolution structural MRI scans and assessments of recent alcohol use and lifetime substance use were collected in a sample of 168 juvenile justice-involved adolescents to explore whether gray matter volumes were associated with past 3-month quantity and frequency of alcohol use. Gray matter volumes were not associated with average quantity of alcohol use. Accumbens volume was positively associated with past 3-month frequency of drinking, and OFC volume was negatively associated with drinking frequency. Results may suggest that structural differences in regions related to reward and control processing may contribute to risk behavior in adolescence.

## 1. Introduction

Altered brain structure and diminished cognitive function are well-established effects of extensive and long-term alcohol use in older adults. Decreased gray matter volumes have been consistently reported in adults with alcohol use disorders (AUD), particularly throughout the frontal cortex and subcortical regions related to reward processing [1,2]. Heavy alcohol use leads to eventual deficits in gray matter volumes, although interactive effects with neural processes in the developing brain are less certain. Significant neural development occurs during adolescence, and differential maturation of neural systems has been hypothesized to underlie risky behavior. Global gray matter volumes typically increase throughout early adolescence as neural connections continue to form before decreasing in middle to late adolescence as excess connections are pruned [3,4]. Reward-seeking subcortical regions develop prior to prefrontal cortical regions involved with executive function and cognitive control [5,6]. This imbalance in reward versus control regions has been hypothesized to be an important precursor of adolescent risk behavior, such that individual differences in brain structure may be associated with increased risk taking including harmful alcohol use.

Functional neuroimaging studies have implicated the nucleus accumbens and frontal regions, particularly the orbitofrontal cortex (OFC), in substance use behavior during adolescence [7,8,9]. One of the neurocognitive mechanisms most often implicated in the initiation and maintenance of substance use behavior is an overemphasis on rewards and discounting of costs or risks associated with obtaining those rewards (*i.e.*, reward bias). Increased activity in the nucleus accumbens has been observed in adolescents relative to children or adults during reward preference tasks [7,10], while activation in the lateral OFC has been associated with selection of larger, delayed rewards [11]. However, despite these findings from functional analyses, few investigations have explored the underlying structure of related regions in adolescent substance users. Studies have suggested that adolescents with an AUD have smaller left hippocampal volumes [12,13], differences in prefrontal cortex white matter volume [14], and smaller cerebellar vermis volume [15]. Given the established roles of the accumbens and OFC in reward processes and existing hypotheses relating differential maturation of these regions to risky behavior, a volumetric approach may elucidate the relationship between substance use and neural development during adolescence. 

The current study tested whether morphometric variables were associated with alcohol use behavior in a large sample of juvenile justice-involved adolescents. In 2009, more than 31 million adolescents were under juvenile court jurisdiction in the United States [16], which represents a non-trivial subsample of adolescents engaging in behaviors with increased risk of negative outcomes. This population may show a wider range of alcohol use than community samples [17] and thus provides a unique opportunity to describe neural correlates of risk behavior. Previous studies of adolescent brain morphometric measures have been limited by relatively small sample sizes and low levels of substance use, and have not examined structural associations related to reward and control systems and recent substance use. The nucleus accumbens was selected as a region of interest due to its involvement in reward processing and demonstrated associations with adult AUD [18,19], while the OFC was selected specifically as a frontal region implicated in substance use behavior [7]. Alcohol quantity and frequency variables were regressed on nucleus accumbens volume, OFC volume, and an accumbens by OFC interaction as well as age and years of substance use to examine the possibility that region volumes may be associated with alcohol use behavior in adolescence. Covariates that may be especially relevant for justice-involved youth, including depression, attention deficit and hyperactivity, and externalizing behaviors, were included in additional models. Given possible structural differences inherent with increasing age and that previous studies of normal brain development have shown dimorphisms between males and females in overall volume [20] and localized cortical and subcortical structures [21,22], age and sex moderators were also examined via interaction terms. 

## 2. Results and Discussion

Alcohol use dependent variables were quantity of alcohol use during a typical dinking occasion in the past 3 months and frequency of alcohol consumption in the past 3 months. Each model also included duration of alcohol use in years (*i.e.*, age of first use subtracted from participant’s current age). Table 1 presents correlations between volumetric and alcohol use variables. Not surprisingly, all alcohol use variables were associated with each other. Volumetric variables were not significantly associated with alcohol use. 

**Table 1:** Correlations between volumetric and alcohol variables.

	1	2	3	4	5	6
1. Accumbens	-					
2. OFC	0.42 **	-				
3. Accumbens by OFC	0.31 **	0.29 **	-			
4. Past 3-month Frequency	0.13	−0.11	0.09	-		
5. Past 3-month Quantity	0.01	−0.09	−0.04	0.55 **	-	
6. Years of Alcohol Use	0.05	−0.02	0.04	0.36 **	0.42 **	-

** *p* < 0.01.

### 2.1. Regression Models

Two sets of multiple regression models explored the relationship between gray matter volumes with quantity of alcohol use over the past 3 months and frequency of alcohol use over the past 3 months. Hierarchical regressions entered age and years of use of alcohol, tobacco, marijuana, and a composite of other drugs in the first step, and volumetric variables for nucleus accumbens, OFC, and nucleus accumbens by OFC interaction in the second step. 

#### 2.1.1. Quantity of Alcohol Use

As can be seen in Table 2, age and lifetime substance use accounted for significant variance in past 3-month quantity of alcohol use [*R*^2^ = 0.21, *F*(5,162) = 8.75, *p <* 0.001]. The volumetric variables added in the second step of the hierarchy did not account for significant additional variability.

#### 2.1.2. Frequency of Alcohol Use

**Figure 1:**
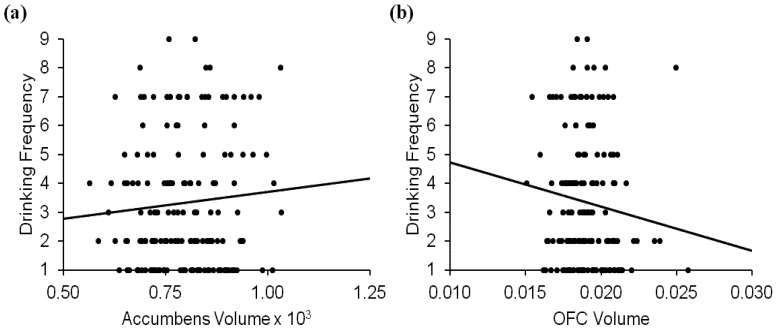
Relationships between (**a**) accumbens volume and past 3-month drinking frequency and (**b**) OFC volume and past 3-month drinking frequency. Volumes are reported as region to intracranial volume ratios.

Age and lifetime substance use also accounted for significant variance in past 3-month frequency of alcohol use (*R*^2^ = 0.15, *F*(5,162) = 5.83, *p <* 0.001, see Table 2). Here, however, volumetric variables added in the second step accounted for significant additional variability (Δ*R*^2^ = 0.05, Δ*F*(3,159) = 2.96, *p <* 0.04). OFC volume was negatively associated (standardized *b* = −0.20, *t*(159) = −2.40, *p <* 0.02) while accumbens volume was positively associated (standardized *b* = 0.18, *t*(159) = 2.16, *p <* 0.04) with past 3-month drinking frequency when controlling for age and lifetime other substance use (see Figure 1). There was no significant accumbens by OFC volume interaction. These results suggest that increased accumbens volume is associated with increased frequency of alcohol use, while increased OFC volume is negatively associated with drinking frequency. 

**Table 2:** Hierarchical regression of past 3-month alcohol use.

	Standardized *b* ^†^	*p*	*R* ^2^	Δ *R*^2^	Δ *F*
*Quantity*					
*Step 1*		*<0.001*	*0.21*	*0.21*	*8.75*
Age	0.06	0.45			
Years Alcohol	0.38	<0.001			
Years Tobacco	−0.16	0.06			
Years Marijuana	0.23	0.02			
Years Other Drugs	−0.05	0.59			
*Step 2*		*0.74* ^‡^	*0.22*	*0.01*	*0.42*
Accumbens	0.03	0.71			
OFC	−0.07	0.38			
Accumbens × OFC	−0.04	0.64			
*Frequency*					
*Step 1*		*<0.001*	*0.15*	*0.15*	*5.83*
Age	−0.06	0.42			
Years Alcohol	0.31	<0.001			
Years Tobacco	−0.10	0.25			
Years Marijuana	0.16	0.10			
Years Other Drugs	0.02	0.88			
*Step 2*		*0.03* ^‡^	*0.20*	*0.05*	*2.96*
Accumbens	0.18	0.03			
OFC	−0.20	0.02			
Accumbens × OFC	0.09	0.25			

^†^ Betas for all predictors are taken from the final model (including Step 2); ^‡^* p*_Δ_.

#### 2.1.3. Possible Moderators of Volumetric Effects on Frequency of Alcohol Use

Given the unique characteristics of this population, the model assessing frequency of alcohol use was examined with an additional step to include covariates for total scale scores measuring symptoms of depression (Children’s Depression Inventory; CDI), attention deficit and hyperactivity (Connors-Wells Self-Report Scale; CASS), and externalizing behaviors (Child Behavior Checklist; CBCL). Not surprisingly, including these covariates accounted for significant additional variance in frequency of alcohol use [Δ*R*^2 ^= 0.06, Δ*F*(3,156) = 4.42, *p <* 0.01]. However, the significance of individual volumetric terms remained relatively unchanged (see Table 3).

**Table 3:** Overall follow-up hierarchical regression models of frequency of alcohol use.

	Standardized *b*	*p*	*R* ^2^	Δ *R*^2^^ ‡^	Δ *F*^ ‡^
*Total Scale Scores*		*<0.01* ^‡^	*0.26*	*0.06*	*4.42*
Age	−0.02	0.77			
Years Alcohol	0.32	<0.001			
Years Tobacco	−0.09	0.29			
Years Marijuana	0.13	0.17			
Years Other Drugs	−0.05	0.59			
Accumbens	0.17	0.03			
OFC	−0.21	<0.01			
Accumbens × OFC	0.11	0.16			
CDI	−0.23	<0.01			
CASS	0.19	0.04			
CBCL	0.11	0.24			
*Moderation by Sex*		*0.47* ^‡^	*0.22*	*0.02*	*0.90*
Age	−0.07	0.34			
Years Alcohol	0.28	0.001			
Years Tobacco	−0.09	0.28			
Years Marijuana	0.16	0.12			
Years Other Drugs	0.04	0.73			
Accumbens	0.08	0.70			
OFC	−0.19	0.26			
Accumbens × OFC	0.22	0.10			
Gender	0.05	0.54			
Accumbens × Gender	0.06	0.74			
OFC × Gender	−0.01	0.98			
Accumbens × OFC × Gender	−0.18	0.08			
*Moderation by Age*		*0.16* ^‡^	*0.22*	*0.03*	*1.77*
Age	−0.09	0.23			
Years Alcohol	0.29	0.001			
Years Tobacco	−0.12	0.18			
Years Marijuana	0.17	0.09			
Years Other Drugs	0.02	0.82			
Accumbens	0.15	0.08			
OFC	−0.20	0.02			
Accumbens × OFC	<0.01	0.93			
Accumbens × Age	0.10	0.29			
OFC × Age	−0.16	0.07			
Accumbens × OFC × Age	0.17	0.13			

^‡^ Change from Step 2 to Step 3 (final model).

Further analyses were run in order to explore moderation of effects by sex and age (see Table 3). Females (*n* = 40) reported fewer years of alcohol use (*t*(166) = −2.03, *p* < 0.05), but not past 3-month frequency of alcohol use, than males (*n* = 128). Both accumbens (*t*(166) = 2.25, *p* < 0.03) and OFC volume (*t*(166) = 4.32, *p* < 0.01) were greater in females. Age was positively associated with years of alcohol use (*r*(166) = 0.21, *p* < 0.01). Similar to the previous model, an additional step was added to include, in one model, the sex and volume by sex interaction terms, and in the second model, the volume by age interaction terms (since the main effect of age was already included in Step 1). No significant overall model changes were observed. Further, no individual interaction terms involving sex reached significance, and the age by OFC volume interaction was only marginal (standardized *b* = −0.16, *t*(156) = −1.81, *p <* 0.08).

### 2.2. Key Findings and Limitations

This study explored the relationships between gray matter volume of the nucleus accumbens and OFC, two regions implicated in reward and control mechanisms of addiction, and quantity and frequency of alcohol use by justice-involved adolescents. Regression analyses attempted to isolate recent use of alcohol by controlling for age and years of alcohol, tobacco, marijuana, and other drug use. Results suggest that volume of the nucleus accumbens and OFC are associated with frequency but not quantity of past 3-month alcohol use. Adolescent studies to date have suggested an association between smaller gray matter volumes in the hippocampus and cerebellum and binge drinking [12,15], as well as an association between smaller accumbens volume and alcohol dependence in adults [18]. However, there have been no previous reports of an association between accumbens volume and substance use in adolescents, nor any reports that examined possible moderators of that relationship. 

Subcortical gray matter volumes typically peak in early adolescence between the ages of 11 and 14 [23], and associations between alcohol use and larger accumbens volume in this sample (14 to 18 years old) may be related to the effect of alcohol on neurodevelopmental processes or preexisting structural differences. Normative loss of gray matter volume in adolescence is likely due to a combination of synaptic pruning, decreasing density, and increasing intra-cortical myelination [24]. Ethanol neurotoxicity may interfere with these processes [25], although it is more likely that the association between gray matter and alcohol use reflects differences that precede or even motivate early alcohol use. For example, it is possible that larger accumbens volume is related to increased dendritic branching as a result of heightened response to reward during alcohol consumption. Research has suggested that alcohol dependent adults show decreased accumbens volume when compared to currently abstinent peers [18], but even heavy adolescent drinkers have limited exposure compared to the decades of neurotoxic exposure found in chronic alcohol-using adults, so that neuronal atrophy may not be a major determinant of regional volumes in adolescent populations. Further, Schneider and colleagues [26] found that adolescents with higher risk-taking bias showed decreased gray matter density in the ventral striatum as well as decreased activation during anticipation of rewards even in the absence of any substance use. These findings suggest that differences in the structure and function of the nucleus accumbens may predispose certain adolescents toward substance abuse [26]. 

Larger OFC volume was associated with decreased past 3-month frequency of alcohol use in this sample. There are very few existing studies that specifically examine the relationship between alcohol use and OFC volume, and none in adolescents. Similar to effects observed for the accumbens, abstinent substance-dependent adults have shown reduced medial OFC volume compared to control participants [27]. Given that cortical gray matter volumes in the frontal lobes typically peak around 10 years of age [23], it might be expected that OFC volume would show a positive association with alcohol use (*i.e.*, if risk behavior is related to delayed neuromaturation in frontal regions, then larger OFC volume would positively correlate with alcohol use). However, the age range of this sample is narrow in the scope of brain development considering that cortical development continues into the early 20s [23]. It is also possible that reduced OFC volume in this sample may be related to neuronal atrophy, as suggested by decreased volumes in other brain regions among adolescents with recent heavy drinking [12,13]. However, our findings are preliminary, and further research is needed to determine whether such neuronal atrophy would differentially target subcortical (*i.e.*, relatively developed by middle adolescence) and cortical regions (*i.e.*, still developing and possibly more sensitive to neurotoxicity).

Previous findings suggest that reward and control systems are involved in risk taking behavior, particularly heavy episodic drinking, in adolescence [7], and the OFC has been implicated in several overlapping brain networks related to addiction in both reward processing and control functions [28]. In the absence of significant interactions, we speculate that decreased OFC volume when controlling for nucleus accumbens volume may be related to increased frequency of drinking. Interestingly, no significant effects of volume on quantity of drinking were observed. Frequency of drinking may provide a better construct of problematic alcohol use in adolescence (*i.e.*, increased reward seeking) than quantity. Quantity of use (*i.e.*, binge drinking) might better reflect a normative pattern of substance use in adolescence. This is consistent with the finding that drinking frequency has shown higher sensitivity and specificity than drinking quantity in identifying adolescents with alcohol-related problems [29]. Alternatively, lack of findings for quantity may also be related to challenges in accurately measuring quantity of alcohol consumption in adolescents. It is also possible that quantity of drinking, and especially loss of control or disinhibition associated with increased heavy episodic alcohol use, may be related to other frontal regions such as the dorsolateral prefrontal cortex. 

One of the primary limitations of this study is the cross-sectional design, such that results are limited to associations rather than predictions. It is important to note that it remains unclear whether differences in gray matter volume precede substance use or whether heavy alcohol use may interfere with important developmental processes related to gray matter volume. The extant literature did not suggest likely hemispheric differences related to the nucleus accumbens or OFC, and so separate right and left volumes were not explored in the current study. It has been suggested that subcortical regions as well as frontal regions may continue to undergo changes through emerging adulthood [30], which may have limited the scope of this study with adolescents 14 to 18 years old. Generalizability to wider adolescent populations is also a concern, given that juvenile justice-involved adolescents are a subset of the general population. However, the current sample represents a wide range of risk behaviors, and volumetric results did not change in the presence of covariates for depression, attention deficit and hyperactivity, and externalizing behaviors. Lastly, this study may have been constrained by sample size, especially in exploratory moderation analyses. 

## 3. Experimental Section

### 3.1. Participants

Justice-involved adolescents (*n* = 225) completed a single session of magnetic resonance imaging and substance use and mood questionnaires as part of a larger study of adolescent risk behavior. Exclusions from the current dataset occurred due to heavy other drug use reported weekly or daily (e.g., frequent cocaine use; *n* = 18), current prescribed psychotropic medication use or bipolar disorder diagnosis (*n* = 22), and listwise exclusion of any missing substance use data (*n* = 17). The final dataset contained 168 adolescents.

The adolescents in this study (76.2% male) were ethnically diverse (61.9% Hispanic/Latino, 14.9% Caucasian, 11.9% Multiracial, 7.1% African American, 2.4% American Indian, 1.2% Asian/Pacific Islander, and 0.6% unknown) with a mean age of 16.51 years (*SD* = 1.10 years). Participants were predominantly recruited from the Youth Reporting Center (YRC), a day program offered by Bernalillo County Juvenile Justice services in Albuquerque, New Mexico. Eligibility criteria for the study included age between 14 and 18 years, proficiency in spoken and written English, and no MRI contraindications (e.g., no irremovable metal implants, not pregnant, not claustrophobic). Both adolescent assent and parental consent via digital audio recording over the telephone were obtained prior to involvement in the study. Participants were instructed that they were not required to answer any questions which made them feel uncomfortable or which they did not wish to answer, and there were minimal instances of missing data. The Institutional Review Board at the University of New Mexico and the federal Office of Human Research Protection approved all aspects of the study, and a certificate of confidentiality was obtained from NIH/NIAAA to protect participants and encourage full disclosure.

### 3.2. Measures

#### 3.2.1. Sample Characteristics

Participants completed common questionnaires regarding their demographics and psychological functioning as part of a larger battery of assessments (see Table 4). The Pubertal Development Scale (PDS) [31] measured self-reported pubertal status through questions regarding specific body changes. Not surprisingly, females (*n* = 39) reported further pubertal development than males (*n* = 128) in this age range, with males closer to midpubertal and females closer to late pubertal categories [*t*(165) = 3.96, *p* < 0.001]. The short form of the Children’s Depression Inventory (CDI-S) [32] was used to assess current symptoms of depression. This 10-item measure describes mood over the past two weeks (e.g., 0 = “I am sad once in awhile”, 1 = “I am sad many times”, 2 = “I am sad all the time”). A suggested cutoff score of 7 indicates clinical depression, and 13% (*n* = 22) of participants met that criterion. Attention deficit and hyperactivity symptoms (e.g., “I have too much energy to sit still for long” or “I bend the rules whenever I can”) were assessed by the Connors-Wells Adolescent Self-Report Scale-Short version (CASS-S) [33] on a 4-point scale (0 = “not at all true” to 3 = “very much true”), with higher raw scores indicating greater symptomatology. The Child Behavior Checklist (CBCL) [34] measured externalizing behaviors through parent report and youth self-report on the rule breaking and aggressive behaviors subscales (e.g., “I don’t feel guilty after doing something I shouldn’t.” 0 = “not true”, 1 = “somewhat/sometimes true” or 2 = “very/often true”). In this sample, 68% of females (*n* = 27) and 56% of males (*n* = 72) exceeded the normed thresholds for clinical levels of externalizing, although raw scores were used in regression analyses since no norms have been recorded for juvenile justice adolescents.

The Rutgers Alcohol Problems Index (RAPI) [35] was used to assess alcohol-related problems. This 23-item measure asks participants to indicate how often problems have occurred as a result of drinking behavior over the last 6 months on a 5-point scale (0 = “never” to 4 = “more than 10 times”). Scores above 20 are generally considered within a clinical range, and 18% (*n* = 30) of participants exceeded that threshold. Participants also completed the Alcohol Use Disorders Identification Test (AUDIT) [36], with summed scores indicating more negative consequences and harmful alcohol use (e.g., regarding occurrence outcomes, 0 = “no”, 2 = “yes, but not in the last 6 months”, 4 = “yes, during the last 6 months”). A score of 13 or more for females or 15 or more for males usually indicates alcohol dependence, and 16% (*n* = 27) of participants met that criterion.

#### 3.2.2. Substance Use Quantity and Frequency

Adolescents completed substance use questions (see Table 4) based on White and Labouvie [35]. Main variables were past 3 month alcohol use frequency (“In the last 3 months, how often did you consume at least one alcohol drink?”) and quantity (“In the last 3 months, how many drinks did you usually have at one time?”). Frequency of use in the last 3 months was rated on a 9-point ordinal scale, with 1 = “never”, 2 = “occasionally”, 3 = “once a month”, 4 = “2–3 times a month”, 5 = “4–5 times a month”, 6 = “once a week”, 7 = “2–3 times a week”, 8 = “4–5 times a week” and 9 = “every day”. Similarly, recent quantity of use was rated 1 = “none”, 2 = “1 drink”, 3 = “2–3 drinks”, 4 = “4–6 drinks”, 5 = “7–9 drinks”, 6 = “10–12 drinks”, 7 = “13–15 drinks”, 8 = “16–18 drinks”, 9 = “19–20 drinks” and 10 = “more than 20 drinks”. This sample reported an average of having 2 to 3 drinks about once a month, although 34 participants reported drinking at least once a week. However, of the 120 participants who reported drinking within the last 3 months, 60% (*n* = 72) reported consuming a typical quantity of 4–6 drinks or more at a time. Duration of substance use was assessed as age of first use subtracted from current age for alcohol, tobacco, marijuana, and other drugs. A composite score was computed for other drug use by coding for the greatest number of years across crack/cocaine, heroin, ketamine, ecstasy, methamphetamines, lysergic acid diethylamide (LSD), gamma-hydroxybutyric acid (GHB), mushrooms, and non-prescribed medications. These variables were used as covariates in regression analyses to better isolate effects of alcohol use.

**Table 4:** Sample characteristics.

Measures	Mean (SD; Range)
*Total Scale Scores*	
Pubertal Development Scale (PDS) ^†^	3.20 (0.39; 1.60–4.00)
Children’s Depression Index (CDI-S)	2.85 (3.17; 0.00–19.00)
Connors-Wells Self-Report Scale (CASS-S) ^‡^	26.99 (13.77; 0.00–74.00)
Child Behavior Checklist (CBCL) Externalizing ^‡^	20.88 (12.78; 0.00–64.00)
Rutgers Alcohol Problems Index (RAPI)	11.66 (13.24; 0.00–92.00)
Alcohol Use Disorders Identification Test (AUDIT)	7.11 (6.93; 0.00–32.00)
*Substance Use*	
Alcohol use frequency (past 3 months)	3.35 (2.25; 1.00–9.00)
Alcohol use quantity (past 3 months)	3.34 (2.22; 1.00–10.00)
Alcohol use (years)	2.69 (2.19; 0.00–11.00)
Tobacco use (years)	1.99 (2.24; 0.00–10.00)
Marijuana use (years)	3.24 (2.49; 0.00–11.00)
Other use (years)	1.16 (1.42; 0.00–6.00)

^†^* n* = 167; ^‡^ raw scores.

### 3.3. Volumetric Data

A 3T Siemens Trio whole body scanner equipped with Sonata gradient subsystem (40 mT/m amplitude, 200 μs rise time, 100% duty cycle) was used to collect MRI images. Structural scans were collected with a multi-echo MPRAGE (MEMPR) sequence with the following parameters: TR/TE/TI = 2300/2.74/900 ms; flip angle = 8°; FOV = 256 × 256 mm; Slab thickness = 176 mm; Matrix = 256 × 256 × 176; Voxel size = 1 × 1 × 1 mm; Number of echos = 5; Pixel bandwidth = 650 Hz; Total scan time = 6 min. This T1 pulse sequence was specifically selected to optimize processing using the FreeSurfer suite, which aligns sequential images based on patterns of cortical folds [37]. After a 3D image of the brain has been reconstructed, an automatic segmentation parcels the brain into distinct regions for volumetric calculations. Complete technical details of parcellation procedures are described by Fischl and colleagues [38,39]. Reconstructions for each participant were visually inspected for accuracy of parcellations. Hand editing was used to establish correlations between edited and fully automated volumes for 10 randomly selected subjects within the sample. In this subset, the correlation between the automated accumbens volume and hand edited accumbens volume was *r* = 0.94, *p* < 0.01. Similarly, the correlation between the automated OFC volume and hand edited OFC volumes was *r* = 0.94, *p* < 0.01. Given this very high correspondence, we utilized the automated volumes in the main analysis. Values for right and left nucleus accumbens were extracted and added for total accumbens volume. OFC values were merged from values for right and left pars orbitalis, medial orbitofrontal cortex, and lateral orbitofrontal cortex [40]. Total intracranial volume was extracted and ratios of region volume to intracranial volume were calculated for use in regression analyses. 

## 4. Conclusions

The current study found significant associations between volume of the nucleus accumbens and OFC and recent frequency of alcohol use in a sample of juvenile justice-involved adolescents, over and above age and lifetime substance use. Results may suggest that structural differences in regions related to reward and control processing may contribute to risk behavior in adolescence. Although it may be premature to suggest that the current findings have wide implications, accumbens or OFC volume may be an important risk marker for alcohol-related problems or the subsequent development of an AUD.

Interestingly, significant results were found for variables related to alcohol use frequency but not quantity. Ideally, future studies should include longitudinal measures of substance use behavior, with careful consideration of increasing validity of both frequency and quantity of use measures. Given that chronic alcohol dependence is associated with reductions in accumbens volume and eventual global reductions in gray matter volume [41], cross-sectional comparisons of late adolescent, young adult, and adult substance users may also prove informative regarding the relationship between structure of subcortical and frontal regions to alcohol or other substance use.

Finally, it is also important to note that while neurodevelopment in these regions may influence adolescent behavior, risky decisions occur within a broader context in which adolescents have the opportunity to take risks [42,43]. The models presented here represent one relatively modest, though significant, piece within a complex overall construct of risk taking behavior. This point may be especially relevant for juvenile justice-involved adolescents who may have greater exposure to risky situations. Juvenile justice-involved adolescents form a large and growing subset of the adolescent population [16], and in many cases it is likely that substance use contributes to or exacerbates problems that initially involve them with the justice system. Improved understanding of the neural correlates of risky behavior, specifically neural structure or mechanisms underlying reward seeking (e.g., structure and function of the nucleus accumbens and OFC) may inform development or improvement of prevention and intervention programs. Further studies should address the possible interactive effects of accumbens and frontal volumes on related risk behaviors or constructs such as impulsivity throughout adolescence, especially in populations with demonstrated risk for long-term negative outcomes.
